# 48842 Sponsor types of US interventional COVID-19 studies listed in ClinicalTrials.gov

**DOI:** 10.1017/cts.2021.499

**Published:** 2021-03-30

**Authors:** Irene Lee, Lisa Cooper, Doreen Waldron Lechner

**Affiliations:** 1 Rutgers, The State University of New Jersey; 2 Department of Health Informatics, School of Health Professions, Rutgers, The State University of New Jersey

## Abstract

ABSTRACT IMPACT: Increase understanding of the types of sponsors responding to the COVID-19 pandemic. OBJECTIVES/GOALS: The COVID-19 pandemic has impacted millions of lives globally. To learn more about this disease and find potential diagnostic, treatment, and preventative products, the healthcare community has initiated a staggering number of clinical trials. Clinicaltrials.gov was reviewed to determine the types of sponsors who are conducting COVID-19 studies. METHODS/STUDY POPULATION: Clinicaltrials.gov was searched using terms ‘COVID-19’ and ‘SARS-Cov-2’. Search results were further defined to include only ‘Interventional’ studies. Of these, only studies with sites located in the United States were selected and for which the ‘Condition’ included at least one of the following terms: ‘COVID’, ‘COVID-19’, ‘Coronavirus’, ‘SARS-Cov-2’, ‘SARS’, or ‘2019-nCoV'. Study sponsors were then categorized as: (1) commercial, (2) academic, or (3) other, based on ‘Sponsor’ information within each study listing. A Google search was conducted for any sponsor that was not easily categorized to obtain additional information to support the proper assessment of sponsor type. The types of sponsors were analyzed over time using the ‘First Posted’ date of each study listing. RESULTS/ANTICIPATED RESULTS: A total of 3662 studies were retrieved, of which 2075 were ‘Interventional’ studies. The studies were further reduced to 681 studies by including only United States sites and the desired ‘Condition’. The percentage of studies from this refined dataset, by sponsor type, were found to be 63% academic, 34% commercial, and 3% other. The relationship between time and sponsor type demonstrated that academic sponsors had the highest percentage of study postings in the first month (March) of the COVID-19 pandemic compared to commercial and other sponsors. Following this first month, academic study postings gradually declined, while commercial sponsors had an increase in postings per month into July, followed by a gradual decline. Few other sponsor type postings were made and occurred primarily in August. DISCUSSION/SIGNIFICANCE OF FINDINGS: The number and timing of listings may be a reflection of study intention and regulatory pathway requirements. Additional variables, such as inconsistent terminology, collaborators, funding, and study start date may influence results. Further analysis may reveal how modification of listing information may result in expedited pandemic response.

